# Applications of Gene-Editing Technologies in Enhancing Crop Stress Resistance with Emphasis on Rice

**DOI:** 10.3390/plants15101476

**Published:** 2026-05-12

**Authors:** Minghui Sun, Fozia Ghouri, Muhammad Waqas, Amjad Ali, Muhammad Azhar Nadeem, Guanqing Wu, Faheem Shehzad Baloch, Muhammad Qasim Shahid

**Affiliations:** 1 State Key Laboratory for Conservation and Utilization of Subtropical Agro-Bioresources, South China Agricultural University, Guangzhou 510642, China; 3118947155@stu.scau.edu.cn (M.S.); foziaghouri@scau.edu.cn (F.G.); wuguanqing@stu.scau.edu.cn (G.W.); 2 Guangdong Provincial Key Laboratory of Plant Molecular Breeding, South China Agricultural University, Guangzhou 510642, China; 3 State Key Laboratory of Cotton Bio-Breeding and Integrated Utilization, Institute of Cotton Research, Chinese Academy of Agricultural Sciences, Anyang 455000, China; mwuaf.edu@gmail.com; 4 Department of Plant Protection, Faculty of Agricultural Sciences and Technologies, Sivas University of Science and Technology, Sivas 58140, Türkiye; amjadbzu11@gmail.com; 5 Department of Biotechnology, Faculty of Science, Mersin University, Yenişehir, Mersin 33343, Türkiye; azharjoiya22@gmail.com (M.A.N.); balochfaheem13@gmail.com (F.S.B.); 6 Department of Biotechnology, Samarkand State University Named After Sharof Rashidov, Samarkand 140104, Uzbekistan; 7 Department of Genetics, Institute of Biochemistry, Samarkand State University Named After Sharof Rashidov, Samarkand 140104, Uzbekistan

**Keywords:** gene editing, CRISPR/Cas9, crop stress tolerance, stress-resistant breeding, genome engineering

## Abstract

Gene-editing technology provides innovative strategies for coping with crop stress, enhancing resistance to biotic stresses (fungal, bacterial, viral infections) and abiotic stresses (salinity, drought, heavy metals, temperature extremes). The CRISPR/Cas9 system is widely used to knock out susceptibility genes, activate resistance genes, or modulate stress-response genes, yielding many stress-resistant crop varieties. However, off-target effects, chimeric effects, and the complexity of multi-gene synergistic editing limit its application. By optimizing and integrating with other cutting-edge technologies, gene editing is expected to yield highly stress-resistant and high-yielding crop varieties, contributing significantly to sustainable agricultural development and ensuring global food security. Rice, a key staple and model plant, has been extensively studied in gene-editing-based research on stress resistance. The practical potential of gene editing for agricultural improvement has been demonstrated by the effective modification of many genes linked to drought, salinity, temperature extremes, and disease resistance using CRISPR/Cas9 and related technologies. This review discusses gene-editing applications in crop stress research, examining the effects of various stresses on crops and the use of gene editing to develop stress-tolerant varieties. It offers substantial guidance for improving crop stress tolerance through gene editing, creating highly resilient cultivars with greater adaptation to complex, variable environments.

## 1. Introduction

**Figure 1:**
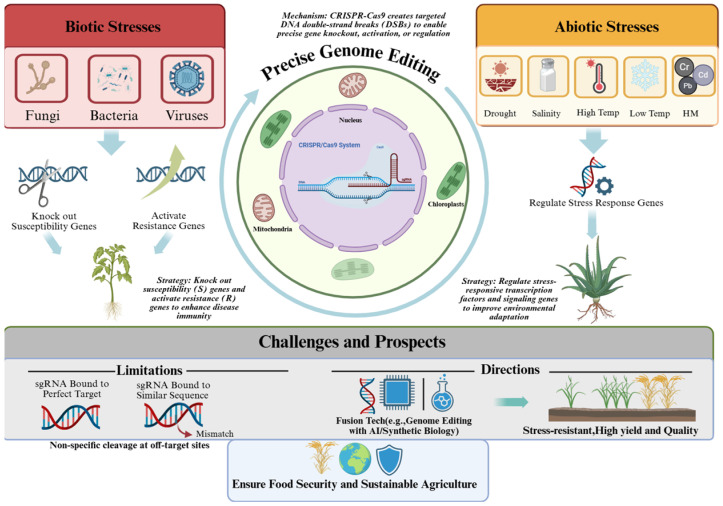
Precise genome editing for improving crop resistance to biotic and abiotic stresses. Temp: temperature; HM: heavy metal.

In recent years, climate change has massively affected crop production, and climate change and food security are interrelated [1,2,3]. Against the backdrop of global climate change, the continuous growth of the worldwide population, and the intensification of climate change [4,5,6], these factors pose unprecedented challenges to crop production [7]. Abiotic stresses such as drought, flooding, high and low temperatures, and salinity, as well as biotic stresses such as pests and diseases, severely affect crop growth, development, and yield [8,9,10,11]. In recent years, according to data from the Food and Agriculture Organization (FAO), about 2% of global crops are lost each year due to adverse stresses caused by climate change [12,13]. This is equivalent to nearly one million hectares of crops being affected each year, leading to a decrease in production. If drought stress leads to soil water deficiency [14], plants cannot obtain enough moisture to maintain normal growth and metabolism, resulting in slowed growth or even cessation [15,16]. Low-temperature freezing damage can cause ice to form inside cells and dehydrate protoplasts, leading to frostbite and the death of crops [17]; pest and disease pressures can directly eat away at or infect crops, leading to reduced yields or even total crop failure [18,19]. Therefore, improving crop stress resistance is imperative. However, using traditional breeding methods to screen for highly resistant varieties presents challenges such as lengthy breeding cycles and the difficulty of simultaneously concentrating multiple genetically controlled desirable traits within a single favorable genetic background. Therefore, gene-editing technology offers a simple and efficient tool for improving crop quality and enhancing stress tolerance. Mutations can be verified by CRISPR-based crop editing [20], followed by sequencing of the target region to confirm the occurrence of the desired mutations, with gene knockout conferring enhanced crop stress tolerance. Alternatively, high-throughput sequencing [21] of edited plants and their wild-type counterparts enables genome-wide analysis of structural variants (SVs), insertions/deletions (InDels), and single-nucleotide variants (SNVs), facilitating assessment of genomic stability post-gene editing. Gene-editing technology, as an emerging genetic engineering technique [22], provides us with a brand new means to directly edit key genes in crops, enhancing their tolerance to various stresses and strengthening global food security [23]. This ensures food security and the sustainable development of agriculture, offers new methods and pathways to address crop stress issues, and shows excellent potential to improve crop resistance. This review systematically summarizes recent research progress in the use of gene-editing technologies to enhance crop stress tolerance. We will focus on case studies of how the identification of key stress-tolerance genes has been applied in stress-tolerance breeding, thereby providing a theoretical framework for leveraging gene-editing technologies to ensure global food security (Figure 1).

## 2. From Traditional Breeding to Gene Editing

**Figure 2:**
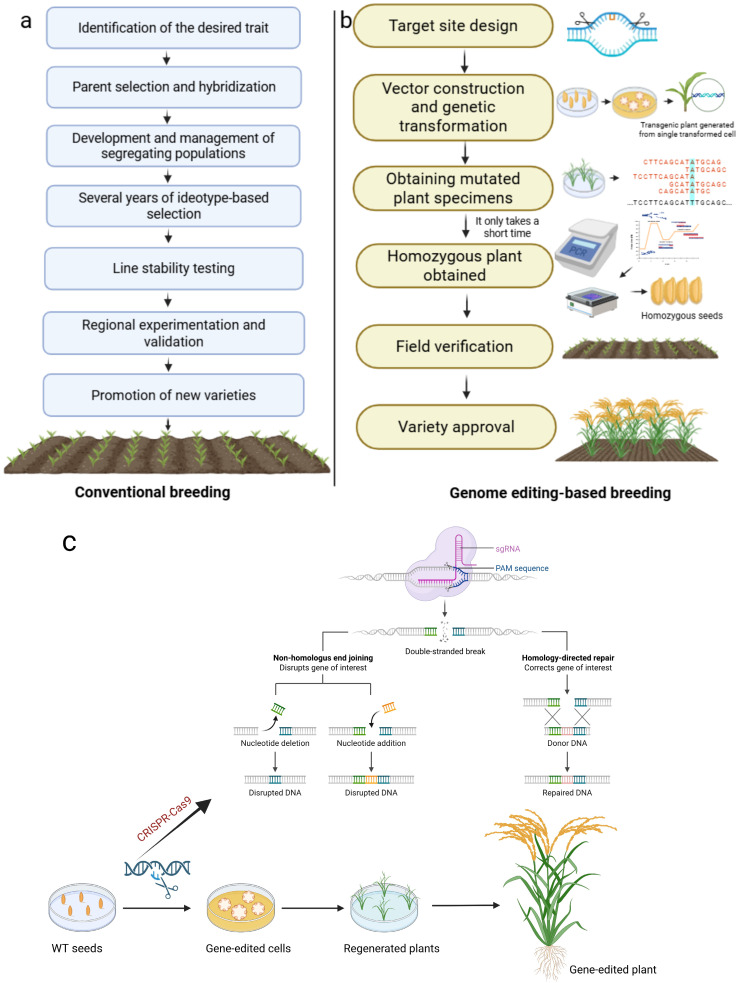
Comparison of conventional breeding and genome-editing-based crop improvement pipelines and the principle of gene editing technology. (**a**) Conventional breeding involves identification of desired traits, parental selection and hybridization, segregation and line development, multi-year selection, stability testing, regional field evaluation, and eventual release of improved varieties. (**b**) Genome-editing-based breeding includes target site design, vector construction and genetic transformation, generation of edited plant lines, selection of homozygous plants, molecular validation, field verification, and final variety approval. The schematic highlights the reduced time and greater precision associated with genome editing approaches compared with conventional breeding. (**c**) The principle and application of gene editing technology to enhance the desired traits. WT: wild type.

Traditional breeding refers to the use of natural variation in biology or artificially created variations, followed by selection and cultivation methods to obtain superior varieties, thereby enhancing the crop’s resilience [24]. Traditional breeding, such as mutation and hybrid breeding, has improved crop yields and played an essential role in the development of agricultural production, providing humans with a wealth of agricultural products and helping ensure food security [25,26]. However, when screening for complex traits controlled by multiple genes, the limitations of traditional breeding become apparent: the screening process is time-consuming and labor-intensive [27], thereby limiting its effectiveness in breeding superior varieties. Breeders select for excellent traits through methods such as hybridization and backcrossing to improve crop yield and quality [28] (Figure 2a). However, because it takes a long time to produce and identify new types with excellent traits, conventional breeding methods have their limits, often requiring several years or even decades to develop a new variety [28,29,30]. Moreover, selection efficiency is relatively low, requiring observation and screening of a large number of descendants to find individuals with the desired traits [31], and it is not easy to select multiple excellent traits simultaneously [32,33].

In addition, traditional breeding methods have relatively limited use of genetic resources and make it difficult to achieve precise improvement of complex traits; it is also difficult to combine desirable traits controlled by multiple genes within a single favorable genetic background [34]. For example, when developing rice varieties resistant to rice blast [35] or when developing high-yielding, disease-resistant, and lodging-resistant corn plants, traditional breeding methods require extensive hybridization and screening to identify parent plants carrying different resistance genes and to integrate their desirable traits into the target varieties [36]. This process is not only time-consuming and labor-intensive but may also introduce undesirable traits through genetic linkage, affecting the yield and quality of rice. However, the emergence of gene-editing technology has provided new approaches and hope for crops to cope with stress. Gene editing, also known as genome editing, is a molecular technology that causes specific changes in the genome by deleting, inserting, or replacing a segment or specific bases [37]. Using this technology, one can precisely target a specific site on the genome, cut the target DNA fragment, and insert a new DNA fragment [38], thereby causing mutations in the gene sequence at that site [38,39] and achieving genetic modification of the DNA sequence. The emergence of gene-editing technology is based on the discovery of Mendel’s laws of inheritance [40], the proposal of the DNA double helix structure [41], and a series of major discoveries, including the discovery of restriction enzymes [42], laying the foundation for gene editing. Compared with traditional breeding methods, gene-editing technology offers advantages such as simplicity of operation, high efficiency, and a short cycle [43]. Through gene-editing technology, scientists can develop crop varieties with specific stress-resistant traits in a short period of time, greatly shortening the breeding cycle [44] (Figure 2b,c). Gene-editing technologies alter plant gene sequences through precise genome modification, including CRISPR/Cas, ZFN, and TALEN technologies [45]. For example, the CRISPR/Cas9 technology [46] can precisely target specific gene loci, like “molecular scissors,” to directly edit DNA sequences, thereby obtaining the desired trait genes.

In contrast, traditional breeding methods can take several years. ZFN and TALEN technologies are early gene-editing tools, but their development has been somewhat limited by high design complexity, high costs, and complex construction processes [47]. Currently, the CRISPR/Cas9 technology has become an important tool for modifying crop genes. It features a simple design, low vector construction cost, and high editing efficiency, and is a commonly used technical method in gene editing [48,49]. In addition, gene-editing technology can break reproductive isolation between species and facilitate cross-species gene transfer, providing richer genetic resources for breeding crops that are resistant to stress. For example, introducing drought-resistant genes from other plants into wheat is expected to result in wheat varieties with enhanced drought resistance.

## 3. Crop Stress

**Figure 3:**
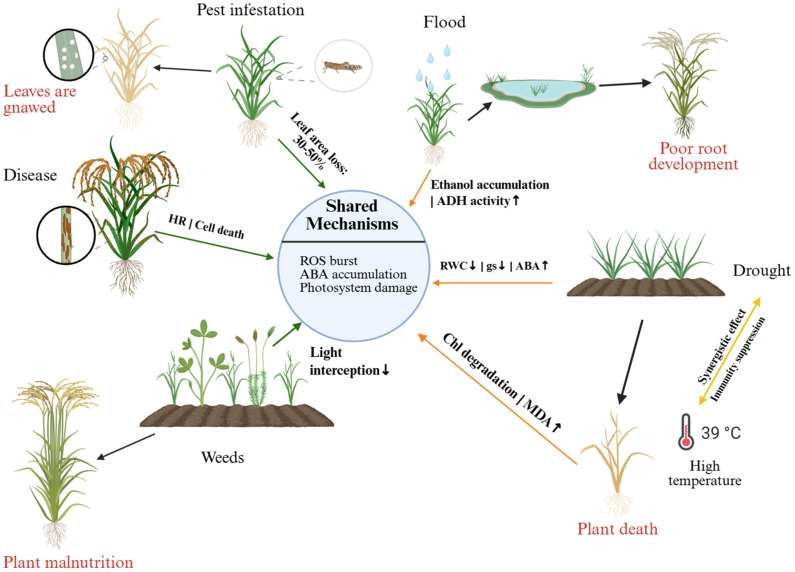
Schematic overview of major biotic and abiotic stresses affecting rice plants and their consequences. Rice plants are subjected to herbivory (leaf gnawing), pest and disease pressure, excess or stagnant water leading to poor root development, nutrient-deficient soils causing plant malnutrition, and high-temperature stress (≈37 °C) that can result in plant death. Arrows indicate the progression from stress exposure to visible growth impairment and yield loss. HR, ROS, RWC, gs, ABA, and ChI represent hypersensitive response, reactive oxygen species, relative water content, stomatal conductance, abscisic acid, and chlorophyll, respectively. ↑: increase; ↓: decrease.

Crop stress refers to a phenomenon in which crops are affected by various adverse environmental factors during growth and development, leading to inhibition or damage of growth, development, physiological functions, and other aspects, thereby affecting crop yield and quality [50,51]. Crop stress is primarily categorized into two major types: biotic stress and abiotic stress [52] (Figure 3).

### 3.1. Biotic Stress

Biotic stress refers to adverse effects on crops caused by other biological factors, generally classified into diseases, insect pests, and weed infestations. These biological factors compete with crops for nutrients, water, and space [53], affecting normal growth and development, reducing yield and quality, and even leading to crop failure or death [50]. Biotic stress not only causes direct economic losses to agricultural production but also negatively impacts the ecological environment.

#### 3.1.1. Diseases

Diseases caused by fungi, bacteria, and viruses are the primary cause of reduced crop yields [54]. When crops are infected with diseases, abnormalities occur in their morphological, physiological, and biochemical characteristics, severely affecting crop yield and product quality [52,53,54,55]. Among these, common diseases in rice include rice blast, bacterial leaf streak, and brown spot [56,57]. Rice blast is the most detrimental, significantly impeding rice plant growth and development, resulting in reduced yields or complete crop failure [58]. Furthermore, wheat rust is a major cause of yield losses in wheat, accounting for up to 20% [59,60].

#### 3.1.2. Pests and Weeds

Weeds and pests in farm fields pose a significant threat to crop growth [61,62,63,64,65]. Excessive weeds in farmland can reduce direct-seeded rice yields by 70–80% [66]. Invasions by herbivorous insects result in a reduction in global agricultural production by about one-fifth each year [67,68]. When weeds grow vigorously in farm fields, they compete with crops for water, nutrients, sunlight, and other resources [69,70], and impede crop growth and development. At the same time, weeds may provide breeding grounds for pathogens and pests [71,72]. Pests such as rice leafhoppers and cotton bollworms feed on crops, hindering their growth and development, and causing significant economic losses to rice and cotton crops each year [73,74,75].

### 3.2. Abiotic Stresses

Abiotic stress denotes the detrimental impacts on crops resulting from abiotic factors, namely, environmental factors, and it also affects interactions between organisms [76]. They are generally adverse effects caused by factors such as drought, high and low temperatures, flooding, and salinization, which have seriously affected crop food security [77,78]. It is predicted that global climate change will increase food demand, while extreme weather will reduce crop yields and nutritional quality [79,80,81]. The damage caused by abiotic stress has far exceeded that caused by biotic stress.

#### 3.2.1. Drought and Flooding

Drought caused by water scarcity is the most severe non-biotic factor affecting global crop yields and the most devastating natural disaster [82,83,84]. During water stress, it affects stomatal closure and inhibits photosynthesis [85,86]. It also disrupts hormonal balance, protein synthesis, and enzyme activity in crops, leading to stunted growth and delayed development [87,88]. The rice root system is extremely vulnerable to water deficit. When the soil water supply is insufficient, the crop roots cannot absorb enough water to meet transpiration and metabolic needs, resulting in water deficiency in the plants [89,90]. At the same time, crop root systems are extremely sensitive to moisture [91]. During periods of water shortage, the upward transport of water from the roots is impeded, severely affecting crop yield and product quality [92,93,94]. On the contrary, in recent years, frequent flooding has severely affected crop yields [95]. Excessive waterlogging reduces oxygen supply to the roots, leading to the accumulation of harmful substances [96]. At the same time, prolonged submersion of the aboveground parts reduces photosynthetic activity [97,98], and impaired respiration ultimately leads to reduced crop yields [99].

#### 3.2.2. Extreme Temperature

In recent years, the frequent occurrence of extreme temperatures (both high and low) has severely impacted agricultural production and threatened food security [100,101,102]. It reduces seed yield by affecting key growth stages of crops (such as the grain-filling stage and the heading and flowering stages), thereby inhibiting the development of floral organs and pollen viability [103,104], resulting in slowed crop growth and development [91,105], growth and metabolic disorders [106,107], heat damage-induced accumulation of ROS, reduced grain-filling rate, lower crop quality, and decreased yield [108,109,110]. In severe cases, the crops die [98].

#### 3.2.3. Salinity and Alkalinity, and Heavy Metal Stress

In addition, soil salinization and heavy metal contamination also pose major challenges to agricultural production [111,112]. Salinity and alkalinity stress trigger a series of physiological and biochemical changes in crops [113,114,115], inhibiting rice growth and nutrient uptake, and reducing grain yield [116]. Salinity stress can also cause iron deficiency in rice, reduce leaf photosynthetic efficiency, and inhibit yield growth [117]. At the same time, salinity and alkalinity stress cause severe damage to the root system, affecting its growth and development [118], and hindering nutrient absorption and the transport of organic matter, leading to reduced yields or plant death [119]. Heavy metals, on the other hand, can directly participate in numerous physiological processes related to plant development and metabolism, including the regulation of enzyme activity and metabolic processes [120,121,122,123,124]. However, when soil heavy metal concentrations exceed the crop tolerance threshold [125,126], these elements can shift from essential to stress factors for crop growth, becoming toxic and posing a threat to crops [127,128,129,130,131]. When soil heavy metal concentrations reach a certain level, they can severely disrupt the soil microbial community and the activity of rhizosphere microorganisms [132]. This triggers a series of stress responses that affect crop growth and development [133]. The accumulation of heavy metals can disrupt crop physiology and damage cellular defense systems by generating excessive reactive oxygen species (ROS) [134,135] and by reducing crop photosynthesis and respiration rates [136]. These effects not only impact crop growth and development but also hinder root growth. Changes in reactive oxygen species negatively impact crop growth and quality [137]. Heavy metals and metalloids markedly impede the growth of polyploid and diploid rice by elevating ROS and altering cellular and antioxidant defense mechanisms [138,139,140,141,142].

In summary, complex and ever-changing biotic and abiotic stresses represent the primary bottlenecks constraining current and future global food security. Traditional breeding methods are characterized by long breeding cycles and low selection efficiency when addressing these stresses. Therefore, the use of emerging gene-editing technologies to precisely modify crop genomes and develop new, high-resistance varieties has become an inevitable choice for ensuring global food security.

## 4. Application of Gene Editing in Biotic Stress

### 4.1. Improvement of Disease-Resistant Genes

Rice blast, caused by the fungus *Magnaporthe oryzae*, is a devastating rice disease known as the “cancer of rice” and is widely distributed in rice-growing regions worldwide. The occurrence of rice blast has a significant impact on the yield and quality of rice, posing a substantial threat to global food security [133]. According to statistics, the annual loss of rice yield caused by rice blast worldwide can be as high as 30–50% [136]. In some severely affected areas, it may even lead to total crop failure [143]. With ongoing advances in gene-editing technology, sequence-specific nucleases (SSNs) are powerful tools for improving crops, and CRISPR/Cas9 is among the most effective. Scientists have used gene-editing tools such as CRISPR/Cas9 to precisely edit genes associated with rice blast resistance, achieving several important results. In recent years, researchers have developed various gene-editing strategies to enhance crop stress tolerance. For example, in the regulation of rice blast disease, *OsGLP2-1* and *OsMESL* represent two distinct regulatory mechanisms.

The germin-like protein (GLP) gene family is a significant defensive gene family that has been documented to participate in plant disease resistance. Studies have shown that *OsGLP2-1*, one of the rice *GLP* genes, is significantly induced by *Magnaporthe oryzae*. To demonstrate the gene’s function, researchers used gene overexpression techniques to precisely regulate its expression. The study found that an increase in rice resistance to bacterial blight, panicle blast, and leaf blast is observed when *OsGLP2-1* is overexpressed [144]. A new disease-resistant mutant gene, methyl esterase-like (*OsMESL*), has been identified in rice, which involves creating functional mutations through gene editing. *OsMESL* affects the accumulation of ROS by interacting with thioredoxin in rice, thereby enabling rice to exhibit substantial tolerance to bacterial blight (*Xanthomonas oryzae*), sheath blight (*Rhizoctonia solani*), and rice blast [145].

In addition, multi-gene-editing strategies are also an important approach in gene-editing technology. For example, maize leaf blight is caused by the fungal pathogen *Setosphaeria turcica*, with lesions primarily appearing on leaves. Infected leaves exhibit wilting and even necrosis, which severely impairs leaf photosynthesis [146,147]. Studies have shown that four genes, namely *Ht1*, *Ht2*, *Ht3*, and *Htn1*, confer resistance to the fungus *Setosphaeria turcica*. Maize inbred lines carrying *Ht2* and *Ht3* were crossed with susceptible parental lines and then backcrossed to develop derived lines. Infected control groups were established, and cultivar specificity was determined by setting up differential groups carrying major *Ht* genes. Finally, it was found that these genes enhance basal defense capacity against pathogens and improve maize stress tolerance [148].

### 4.2. Research on Herbicide Resistance

Due to excessive weed growth in farmland, herbicide applications may affect crop growth and development, or even cause crop death. Therefore, it is necessary to use gene-editing tools—particularly the CRISPR/Cas9 system—to develop herbicide-resistant (HR) crops to eliminate weeds and increase crop yields [149]. The CRISPR/Cas9 technology can be used to research herbicide-resistant crops [150]. Zhang et al. found that, through precision editing of the *SH4* and *qsh1* genes, which control grain shattering in rice, and by introducing specific genetic mutations, the genetically modified weedy rice lines exhibited a significantly lower tendency to shatter their seeds, thereby reducing yield losses [151]. Second, for herbicides such as glyphosate, which are lethal to both weeds and major crops, targeted base editing is achieved through CRISPR/Cas9-mediated homology-directed repair (HDR), which introduces base substitutions into endogenous genes in rice. To address the sensitivity of crops to glyphosate, gene editing was employed to generate T2-edited plants containing *mCcEPSPS*, which were studied. These plants demonstrated persistent inheritance of mutations, reduced glyphosate-binding affinity, and retained optimal photosynthetic and agronomic characteristics after glyphosate application, thereby enhancing crop stress resistance.

### 4.3. Research on Insect Resistance

Pest infestations are one of the main factors contributing to reduced global food crop yields. CRISPR/Cas9 technology can alter insect DNA to trigger gene mutations or combat insect resistance to specific insecticides [152]. For example, *Spodoptera frugiperda* is an extremely destructive agricultural pest [153]. Since *dsx* regulates sexual differentiation in insects, researchers used CRISPR/Cas9 technology to target this gene precisely and found that adult male pests with *dsx* mutations are unable to mate with other males, thereby reducing the pest population. This represents an effective pest control method [154]. Meanwhile, the promoters or other regulatory regions of insect resistance-related genes in crops can be targeted for disruption, substitution, or modification of key cis-acting elements within the promoter, thereby finely tuning their expression levels and patterns and enhancing crop insect resistance.

Research has found that the expression of trypsin inhibitor genes in rice increases when the plant is infested by pests, thereby inhibiting pest growth by suppressing enzyme activity within their bodies. By using CRISPR/Cas9 technology to precisely modify the promoter region of the pest-resistant gene, researchers have significantly inhibited the growth and development of pest larvae, thereby reducing the damage they cause [155].

## 5. Application of Gene Editing in Abiotic Stress

### 5.1. Research on Drought Resistance

Due to climate change, drought has severely impacted rice growth, development, and yield worldwide [156]. Researchers are employing a variety of research and development strategies to address the risks posed by climate-induced droughts.

The *OsMYBR1* gene was identified by investigating MYB proteins. Plants overexpressing *OsMYBR1* were produced using gene editing and subjected to drought tolerance investigation by assessing various physiological indicators, including free proline, soluble sugar concentrations, and abscisic acid (ABA) sensitivity, in comparison to wild-type plants. The results demonstrated that overexpression of *OsMYBR1* resulted in a buildup of soluble sugars and free proline, reduced susceptibility to abscisic acid, and thereby enhanced drought resistance in plants [157]. It primarily responds to drought stress by altering physiological indicators. In addition, a gene, *OsWIH2*, was identified that potentially participates in the induction of plant drought resistance. Using Nipponbare as the genetic background, they generated *OsWIH2*-overexpressing (*OsWIH2-OE*) and RNA interference (*OsWIH2-RNAi*) lines. Subsequent drought treatment and rehydration assays revealed that compared to *OsWIH2*-RNAi and WT plants, the survival rate of *OsWIH2*-OE plants under drought stress was substantially greater. Moreover, *OsWIH2-OE* plants exhibited reduced ROS accumulation, thereby enhancing their resistance to drought stress [158]. This gene primarily focuses on post-stress recovery. In contrast, Liu et al. investigated the role of *Osgf14b* in rice drought tolerance. Compared to the wild-type, *osgf14b* mutants altered the expression of multiple stress-related genes, making them more resistant to drought and osmotic stress. *osgf14b* mutants enhanced crop drought tolerance, whereas *OsGF14b* overexpression lines were more sensitive to drought stress [159]. This reminds us that, for certain genes, CRISPR/Cas9 knockout may confer greater resistance than overexpression. For example, Ye et al. used CRISPR/Cas9 to modify the *Rc* gene, thereby eliminating the harmful husk trait in wild rice and improving the drought tolerance of its seeds during germination [160]. Furthermore, in an experimental environment simulating drought conditions, the yield of gene-edited rice increased by more than 30% compared with the unedited control. The miR1432-*OsCaML2* module studied by Luo et al. fine-tunes downstream target genes by regulating microRNAs; miR1432 influences rice drought tolerance by directly targeting the *OsCaML2* gene [156] (Table 1).

### 5.2. Research on High-Temperature Tolerance

Using CRISPR/Cas9 gene-editing technology, researchers introduced a heat-stable Rubisco activase into *Arabidopsis* and into heat-adapted wild rice species, either by targeted knock-in of the heat-stable gene or by replacing the endogenous Rubisco activase gene. This precise gene-editing approach increased Rubisco activase activity, boosting photosynthesis and promoting plant growth under high temperatures. Recently, significant progress has been made in elucidating the biochemical, physiological, and molecular mechanisms of heat tolerance using gene-editing technology. In terms of the stress response, heat shock proteins (HSPs) are the first line of defense against heat stress in plants. For example, Hsp101, BOBBER1, and Hsa32 have been shown to be important for inducing and maintaining heat tolerance. The downstream target genes and upstream regulatory factors of *HsfA2*, including *Hsa32* and *Apx2*, as well as heat shock transcription factor-binding proteins, are involved in regulating heat tolerance [161].

In contrast, Xiao Langtao’s team’s research on *RARE1* has revealed another heat-tolerance strategy. The study unexpectedly found that plants exhibited pronounced high-temperature intolerance after knocking out the *RARE1* gene. Further studies revealed that both the expression of the *RARE1* gene and the corresponding editing efficiency of *accD* respond to temperature changes. Artificially increasing the editing efficiency of *accD* in plants could significantly enhance their high-temperature tolerance, indicating a positive correlation between *accD* editing efficiency and plant high-temperature tolerance. In-depth investigations showed that changes in *accD* editing efficiency directly affect the activity of acetyl-CoA carboxylase (ACCase), a key enzyme in fatty acid synthesis, thereby regulating the production of unsaturated fatty acids and impacting how well biological membranes withstand elevated temperatures. This study not only comprehensively uncovers the molecular mechanism by which RNA editing of the chloroplast *accD* gene in plants participates in regulating ACCase activity and fatty acid synthesis to respond to high-temperature stress, but also expands the theoretical understanding of RNA editing under stress conditions, and has important guiding significance for molecular plant breeding [162]. Researchers focused on the *PIF7* gene in *Arabidopsis thaliana*, a *bHLH* transcription factor that plays a crucial role in the transcriptional regulatory mechanism in response to high-temperature stress [163].

### 5.3. Research on Submergence Tolerance

Prolonged submergence leads to hypoxia in plants, which impairs their growth and development. Plants have evolved two opposed survival strategies in response to waterlogging stress: the “elongation strategy” and the “stasis strategy.” A differentially expressed gene, *HvERF62,* was identified via a genome-wide association study (GWAS) on barley populations. CRISPR/Cas9-mediated knockout mutants of this gene exhibited sensitivity to submergence, reduced adventitious root formation, and decreased chlorophyll content. This gene regulates stomatal formation in plant tissues and ROS homeostasis [164]. Among these, the flood-tolerant locus *Sub1A-1* represents the dormancy strategy, while the floating genes *SNORKEL1 (SK1*) and *SNORKEL2 (SK2)* represent the elongation strategy, regulating different flood responses in rice [165]. Varieties carrying *Sub1A-1* exhibit restricted elongation and carbohydrate conservation, whereas *SK1*/*SK2* promote rapid internode elongation, together contributing to improved survival under submergence stress. The submergence responses in rice are hormonally regulated: *SUB1A-1* promotes quiescence by suppressing gibberellin-mediated elongation, while *SK1/SK2* enhances internode elongation through ethylene–GA signaling, thereby improving survival under flooding stress [166].

Gammanpila et al. [167] reported that hypoxia-responsive transcription factors, particularly *SUB1A* and *AP2*/*ERF* family members, regulate anaerobic metabolism, conserve energy, and improve rice survival under flooding stress by inhibiting elongation growth while submerged. Interestingly, indigenous rice landraces were screened for the submergence tolerance loci *Sub1A* and *SNORKEL* (*SK1*/*SK2*), and it was found that several genotypes simultaneously carried both loci, enabling either quiescence or elongation strategies and thereby enhancing survival under mixed flooding conditions [168]. In addition, the CRISPR/Cas9 gene-editing system was used to generate a targeted knockout mutant of the *SAB23* gene in rice. This mutant, along with overexpressed lines and wild-type plants, was subjected to submergence assays to investigate the gene’s function. The results showed that under submergence stress, the *SAB23* knockout mutant exhibits significantly reduced endogenous GA4 levels, resulting in stunted growth, delayed seed maturation, and reduced seed set. Thus, *SAB23* regulates submergence tolerance in rice by mediating GA_4_ levels.

### 5.4. Research on Salt-Alkali Tolerance

One salt stress-responsive gene, *OsSalT*, was found on rice chromosome 1 QTL. The findings linked *OsSalT* to the plant adaptive stress response and demonstrated that it operates via pathways that are dependent on abscisic acid and gibberellic acid. When it is overexpressed in plant models, they become more resistant to drought and salt. Additionally, plants that have been transformed with *OsSalT* show improved seed germination, earlier flowering, and stronger root growth. Using the elite three-line restorer gene R192 as the recipient in breeding, CRISPR/Cas9 was employed to target *OsRR22*, a major gene controlling salt tolerance in rice. To determine the seedlings’ salt tolerance, they were exposed to 0.4% and 0.8% NaCl solutions when they had three leaves. The study’s results establish a standard for future efforts to increase rice’s salt tolerance; specifically, the novel germplasm with an *OsRR22* mutation produced by CRISPR/Cas9 shows promise in this regard [169].

**Figure 4:**
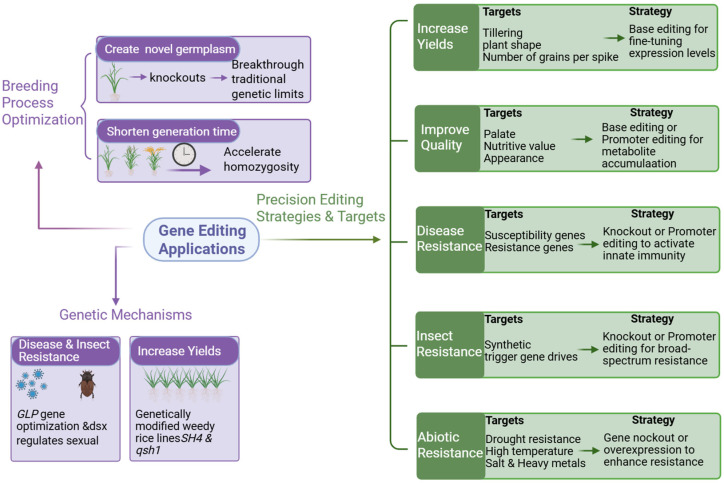
Applications of gene editing in crop improvement.

The WRKY family of transcription factors is highly prominent in plants. Along with controlling plant development and growth, it also participates in regulating plants’ ability to deal with biotic and abiotic stressors. The *PtWRKY39*-overexpressing transgenic lines showed drought resistance and salt-alkali tolerance at the germination and seedling stages. Under root irrigation stress, phenotypic analysis demonstrated that transgenic lines exhibited substantially superior growth compared to the wild type (WT) in transgenic seedlings with seven leaves subjected to saline-alkali soil extract and sodium chloride treatment. The results of this work show that transgenic plants can better regulate their resistance to drought, salt, and alkali when the *PtWRKY39* gene is overexpressed during vegetative growth. The *AT1* gene plays a vital role in rice’s salt-alkali tolerance mechanism. Studies have found that when crops are damaged by salt and alkali, cells will produce ROS. An appropriate amount of ROS can act as signal molecules to regulate metabolic reactions, such as plant stomatal closure and disease defense. However, excessive accumulation disrupts the balance of reactive oxygen species in crop cells, affecting crop growth and development. Aquaporins can “pump” excess reactive oxygen species inside cells to the outside of cells, and only phosphorylated aquaporins have this function. The *AT1* gene inhibits the phosphorylation of aquaporins; knocking out *AT1* through gene editing can enhance rice salt tolerance [170,171] (Figure 4).

Gene editing expedites breeding by reducing breeding cycles and facilitating the development of novel germplasm. These applications promote crop traits, including yield enhancement and quality improvement, while bolstering crop resilience by boosting resistance to diseases, pests, and insects, as well as abiotic stresses.

### 5.5. Research on Heavy Metal Tolerance

In recent years, due to land pollution, an increasing number of heavy metals have accumulated in soil, exerting a range of toxic effects on crops. Breeders have explored stress-resistant genes through gene editing, such as CRISPR-Cas9, to perform targeted knockouts, replacements, or regulation of gene expression involved in heavy metal tolerance, mitigating the effects of heavy metals on crops [171]. Zhou et al. used CRISPR-Cas9 to edit the wheat epigenome, thereby enhancing its tolerance to heavy metal stress and elucidating the gene functions underlying its response to heavy metal stress [134]. The effects of cadmium (Cd) stress on rice growth were investigated using wild-type rice and *Osnramp5* mutant rice generated via CRISPR/Cas9 technology. Arbuscular mycorrhizal fungi (AMF) can regulate the absorption of heavy metal ions by plant roots. The study found that Cd treatment upregulated the expression of the *OsNRAMP5* gene in rice leaves. Notably, inoculation with *Rhizophagus irregularis* (Ri) significantly inhibited Cd translocation from rice roots to leaves, which may be attributed to AMF-mediated modulation of heavy metal transport. Additionally, the Cd content in *Osnramp5* mutants was significantly lower than that in wild-type rice, indicating that the *OsNRAMP5* gene is a key regulator controlling Cd ion transport in rice [172] (Table 1). This gene-editing approach, which involves targeted gene knockout, effectively reduces cadmium accumulation in crops. In addition, the *OsNRAMP* family plays a crucial role in rice tolerance to heavy metal stress. Studies have shown that *OsNRAMP1* mutants enhance crop tolerance by regulating ROS and heavy metal ion transport [173]; the *OsNRAMP2* mutant regulates the transport of heavy metal ions to the aboveground parts of the plant, thereby reducing their accumulation in the grains [174]; and *OsNRAMP3/4/6/7* contribute to stress resistance by regulating the homeostasis of trace elements [175].

The *OsHMA* family serves as a key regulatory hub in crop responses to heavy metal stress. Research findings indicate that *OsHMA2* mutants can transport both the essential nutrient zinc and the toxic heavy metal cadmium, thereby promoting the translocation of cadmium to the aboveground parts of the plant [176]; in contrast to the transport function of *OsHMA2*, *OsHMA3* serves as the first line of defense against cadmium toxicity, preventing its upward transport [177]; functional loss mutations in the *OsHMA4* gene lead to increased copper transport into the grains, resulting in excessively high copper levels in the grains [178]; and overexpression of *OsHMA5* significantly increases copper transport to the aboveground parts, whereas knocking out *OsHMA5* reduces copper content in the aboveground parts [179].

Heavy metals are absorbed by plants in ionic form at the roots and translocated upwards. Rapeseed (*Brassica napus*) is considered a promising candidate for phytoremediation due to its ability to accumulate high concentrations of heavy metals [180]. Zhang et al. performed targeted knockout of two homologous copies of the *BnABCG36* gene (a PDR family member) in rapeseed using CRISPR-Cas9. The study found that the knockout mutants exhibited enhanced cadmium (Cd) ion absorption, resulting in a significant increase in shoot Cd accumulation compared with the wild type. Therefore, *BnABCG36* is involved in Cd ion efflux, and its knockout leads to a substantial accumulation of Cd ions in the shoots [180].

Studies have shown that sucrose transporters (*SUTs*) significantly impact plant responses to various forms of abiotic stress [181]. Zheng et al. employed *OsSUT2*/*OsSUT4* double mutants to investigate the impacts of these two genes on sucrose transport under cadmium stress. Stress treatments were conducted on *OsSUT2* and *OsSUT4* knockout mutants, followed by the determination of differences in sugar metabolism. The results demonstrated that CRISPR/Cas9-mediated targeted knockdown of sucrose transporters *OsSUT4* and *OsSUT2* reduces rice resistance to cadmium stress by disrupting sugar transport, suppressing chlorophyll accumulation, elevating ROS levels, and lowering antioxidant defenses [182].

**Table 1:** Summary of key stress-responsive genes in major crops.

Gene	Crop	Stress	Main Effect	Reference
*OsGLP2-1*	Rice	Disease	Enhances resistance to bacterial leaf blight, panicle blast, and leaf blast	[144]
*OsMESL*	Rice	Disease	*OsMESL* interacts with thioredoxin in rice to affect ROS accumulation, conferring considerable tolerance to bacterial leaf blight, sheath rot, and rice blast.	[145]
*SnRK1β1A*	Rice	Disease	Negatively regulates rice’s innate immunity and is a common target of conserved effector proteins from various fungi	[183]
*ERF922*	Rice	Disease	Negatively regulates disease resistance pathways; knockout results in increased resistance	[184]
*OsMads26*	Rice	Disease, Drought	Negative regulation of rice disease resistance and drought tolerance	[185]
*OsJAZ10*	Rice	Disease	Through a specific frameshift mutation, a new protein is produced that promotes lignin synthesis and strengthens cell walls, thereby enhancing insect resistance.	[186]
*Ht1, Ht2, Ht3, Htn1*	Maize	Disease	Enhances basal defense capability against pathogens	[148]
*ZmMYB74*	Maize	Disease	Affects stalk rot resistance by negatively regulating the key lignin synthesis gene *ZmCAD*	[187]
*Lr34* *, Lr67*	Wheat	Disease	ABC transport protein changes membrane permeability	[188]
*CERK1-V*	Wheat	Disease	Improved resistance to powdery mildew, yellow rust, and *Fusarium* wilt	[189]
*TaROD1*	Wheat	Disease	Improves the resistance of wheat	[190]
*OsCaML2*	Rice	Drought	miR1432 affects drought resistance in rice by directly targeting the *OsCaML2* gene.	[156]
*osgf14b*	Rice	Drought	The *osgf14b* mutant alters the expression of stress-related genes, making it more resistant to drought and osmotic stress.	[159]
*OsMYBR1*	Rice	Drought	Overexpression of *OsMYBR1* leads to the accumulation of soluble sugars and free proline, and reduces sensitivity to abscisic acid, thereby enhancing plant drought resistance.	[157]
*OsWIH2*	Rice	Drought	*OsWIH2*-OE plants show reduced ROS accumulation, thereby enhancing their resistance to drought stress.	[158]
*OsDIS1*	Rice	Drought	Encodes the E3 ubiquitin ligase; its knockout reduces stomatal density and optimizes root architecture, thereby improving water use efficiency	[191]
*OsNAC092*	Rice	Drought	After knockout, the plants exhibited drought sensitivity, whereas overexpression enhanced drought tolerance.	[192]
*OsPP2C03*	Rice	Drought	Following gene knockout, stomatal regulation is impaired, and the plants become more sensitive to drought.	[193]
*OsDUF2488*	Rice	Drought	Overexpression of this gene significantly enhances rice tolerance to water stress.	[194]
*OsRAV11*	Rice	Drought	The survival rate of the knockout line under drought stress is significantly higher than that of the wild type.	[195]
*ZmASR1*	Maize	Drought	The *ZmASR1* knockout lines show lower ROS accumulation, higher ABA content, and greater stomatal closure than wild-type plants, thereby exhibiting greater drought tolerance.	[196]
*ZmC2DP1*	Maize	Drought, Salinity	Knocking out *ZmC2DP1* enhances resistance to drought and salt stress.	[197]
*ABP2*	Maize	Drought, Salinity	*ABP2* reduces ROS levels in transgenic plants.	[198]
*ZmVPP1*	Maize	Drought	Survival rate of transgenic seedlings increases under drought stress.	[199]
*TaLBD16*	Wheat	Drought	Enhances drought resistance by activating key genes in the ABA signaling pathway	[200]
*TabZIP8-7A*	Wheat	Drought	Improves plant drought resistance and ABA sensitivity	[201]
*HsfA2*	Rice	Heat	Enhances heat tolerance in rice	[161]
*RARE1*	Rice	Heat	Expression of the *RARE1* gene and corresponding *accD* editing efficiency respond to temperature changes; improving plant *accD* editing efficiency can significantly enhance heat tolerance.	[162]
*GS3*	Rice	Heat	The *gs3* mutant exhibits greater heat tolerance during the heading stage and has a higher relative grain-filling rate under high temperatures.	[202]
*OsMFT1*	Rice	Heat	Knocking out *OsMFT1* maintains the structural integrity of chloroplasts and significantly enhances the heat tolerance of rice seedlings.	[203]
*OsRbohB*	Rice	Heat	The enzyme encoded by this gene causes excessive accumulation of reactive oxygen species (ROS) at high temperatures, leading to cell damage. Knocking out *OsRbohB* significantly reduces ROS-induced damage to plants.	[204]
*ZmCDPK7*	Maize	Heat	Located in the cytoplasmic membrane, it transfers from the membrane to the cytoplasm at high temperatures, increasing heat resistance.	[205]
*ZmHUG1*	Maize	Heat	The knockout mutant of *ZmHUG1* shows significantly increased sensitivity to heat stress.	[206]
*TaHsfA2-1*	Wheat	Heat	*TaHsfA2-1* improves plant heat tolerance by mediating Hsps expression.	[207]
*TaHsfC2a*	Wheat	Heat	Overexpression of *TaHsfC2a* significantly enhances heat stress tolerance, while knockout significantly reduces tolerance.	[208]
*SUB1A-1*	Rice	Flooding	Promotes quiescence by suppressing gibberellin-mediated elongation, improving the survival rate	[166]
*SNORKEL*	Rice	Flooding	This gene enhances internode elongation through ethylene-GA signaling, thereby improving survival under flooding stress.	[165]
*SAB23*	Rice	Flooding	*SAB23* regulates submergence tolerance in rice by modulating GA4 levels.	[168]
*NAL11*	Rice	Flooding	Knockout promotes ABA accumulation, reduces GA synthesis, stimulates meristem elongation, and simultaneously enhances the antioxidant enzyme system to withstand waterlogging stress.	[209]
*ZmEREB180*	Maize	Flooding	Reduces flooding stress by enhancing adventitious root formation and regulating antioxidant levels	[210]
*HvERF62*	Barley	Flooding	This gene regulates stomatal formation and ROS homeostasis in plant tissues.	[164]
*OsSalT*	Rice	Salinity	This gene functions through abscisic acid- and gibberellin-dependent pathways.	[169]
*OsRR22*	Rice	Salinity	This gene is a major regulatory gene for salt tolerance in rice.	[169]
*PtWRKY39*	Rice	Drought, Salinity	Overexpressing transgenic lines show drought resistance and salt-alkali tolerance during germination and the seedling stages.	[170]
*AT1*	Rice	Salinity	*AT1* gene inhibits phosphorylation of aquaporins; knockout of *AT1* through gene editing can enhance salt tolerance in rice.	[170,171]
*OsNAC10*	Rice	Salinity	Enhances the recycling of Na^+^ in the aboveground parts	[211]
*HST1*	Rice	Salinity	Reduces Na^+^ accumulation and increases proline content and antioxidant enzyme activity	[212]
*STRK1*	Rice	Salinity	Negatively regulates salt tolerance; knockout enhances H_2_O_2_ homeostasis and antioxidant capacity	[213,214]
*OsAld-Y*	Rice	Salinity	Enhances tolerance to alkaline stress during the seedling stage	[215]
*ZmHAK4*	Maize	Salinity	Absorbs Na^+^ from the xylem into the surrounding parenchyma cells, reducing the transport of Na^+^ from the root to the aerial parts	[216]
*ZmHAK1* *1*	Maize	Salinity	Affects salt-alkali tolerance by reducing Na^+^ transport from corn roots to the aerial parts	[217]
*TaHKT1;5-D*	Wheat	Salinity	Expels Na^+^ from the parenchyma cells in the xylem and reduces the transport of Na^+^ to the aboveground parts	[218]
*TaSOS1*	Wheat	Salinity	Pumps excess Na^+^ out of the cell to reduce cytotoxicity	[219]
*OsNRAMP5*	Rice	Heavy Metal	Regulates heavy metal ion uptake by plant roots	[172]
*OsNRAMP1*	Rice	Heavy Metal	Involved in Cd transport and plant immune regulation	[173]
*OsNRAMP2*	Rice	Heavy Metal	Reduces transport to the aboveground parts and decreases accumulation in the grains	[174]
*OsNRAMP3/4/6/7*	Rice	Heavy Metal	Regulates the homeostasis of essential trace elements and enhances rice stress tolerance	[175]
*OsHMA2*	Rice	Heavy Metal	Participates in the loading of xylem from the roots to the aboveground parts	[176]
*OsHMA* *3*	Rice	Heavy Metal	Inhibits the transport of Cd to the aboveground parts	[177]
*OsHMA* *4*	Rice	Heavy Metal	Excess copper ions are pumped into vacuoles for storage, thereby preventing their upward transport and subsequent excessive accumulation in the grains.	[178];
*OsHMA5*	Rice	Heavy Metal	Involved in the regulation of copper ion transport	[179]
*OsSUT2/OsSUT4*	Rice	Heavy Metal	Knockout of sucrose transporters *OsSUT4* and *OsSUT2* reduces rice resistance to cadmium stress by interrupting sugar transport, inhibiting chlorophyll accumulation, elevating ROS, and decreasing antioxidant defense capacity.	[182]
*OsLCD*	Rice	Heavy Metal	Cadmium levels in the grains were significantly reduced following the knockout.	[220]
*OsLSi1*	Rice	Heavy Metal	Knockout reduces silicon uptake in rice.	[221,222]
*OsZIP1*	Rice	Heavy Metal	“Pumps out” or “sequesters” excess zinc, copper, and cadmium ions from the cytoplasm, thereby reducing the toxicity of these metals to the cells	[223]
*OsIRT1/2*	Rice	Heavy Metal	Overexpression of *OsIRT1* significantly increases the iron and zinc content in rice grains.	[224]
*ZmHIPP27*	Maize	Heavy Metal	Differential expression in maize roots under cadmium toxicity	[225]
*TaHSP17.8*	Wheat	Heavy Metal	Compared with wild-type (WT) plants, the transgenic lines showed reduced damage to both root and shoot tissues.	[226]
*BnABCG36*	Rapeseed	Heavy Metal	*BnABCG36* is involved in Cd ion efflux; knockout of this gene leads to massive accumulation of Cd ions in stems.	[180]

## 6. Future Outlook

Against the backdrop of global climate change and continued population growth, ensuring food security and sustainable agricultural development has become a major challenge for humanity [227]. As a key breakthrough in modern biology, gene-editing technology offers a novel solution for addressing biotic and abiotic stresses faced by crops, with extensive application prospects. Gene-editing technology is constantly evolving. On one hand, editing tools are becoming increasingly diverse, precise, and efficient. The CRISPR/Cas system can recognize different PAM sequences, expanding the targeting range of gene editing. Base editing technology enables precise alterations of single or multiple bases in genes, markedly enhancing editing accuracy while minimizing unintended effects on adjacent genomic regions. The integration of gene editing with other cutting-edge technologies is improving. When integrated with artificial intelligence, it can utilize AI’s advanced data analysis and predictive abilities to efficiently identify and design optimal gene-editing targets, minimize off-target effects as much as possible [228], simulate the effects of editing, and shorten the research and development timeline. For example, computer-based tools can be used to predict gRNA cleavage efficiency, eliminate unnecessary target genes, and shorten the R&D cycle [229]. By identifying patterns of gene expression, the machine can predict which genes will be expressed in response to stress, thereby helping the organism cope with the stress [230]. The intersection with synthetic biology enables the design and construction of entirely new biological components and regulatory networks from scratch, allowing for more complex and precise editing of crop stress resistance. This includes creating new stress response regulatory modules and introducing them into crop genomes.

From the perspective of applications in crop stress resistance breeding, gene-editing technology is expected to cultivate a large number of new crop varieties with multiple resistances and wide adaptability. For biotic stresses, it can precisely knock out or modify susceptibility genes in crops that are exploited by pathogens, enabling them to acquire durable resistance to multiple pathogens. Meanwhile, by activating or overexpressing the crop’s own resistance genes, the crop’s immune system can be enhanced and its resistance spectrum expanded, allowing crops not only to resist common diseases and pests but also to have a certain defense capability against newly emerging or mutated pathogens. In the face of abiotic stresses, gene editing can be used to regulate key genes in crops involved in stress response pathways, including drought, salinity, and high and low temperatures. For example, it can regulate the expression of genes related to osmotic adjustment substance synthesis, antioxidant enzyme genes, ion transporter genes, etc., to breed crop varieties that can efficiently utilize water in arid areas, grow normally in saline-alkali land, and maintain yields under extreme temperatures. Moreover, in the future, it may be possible to simultaneously edit multiple genes conferring different forms of stress resistance, enabling crops to exhibit comprehensive stress resistance and adapt to more complex, variable environments.

In the field of the ecological environment, gene-editing technology will also play a positive role. On the one hand, it can reduce agricultural production’s dependence on chemical pesticides and fertilizers, thereby lowering agricultural pollution. By cultivating crop varieties resistant to diseases, pests, and abiotic stresses, the use of pesticides and fertilizers can be reduced, thereby alleviating their pollution of soil, water, and air and protecting the ecological balance and biodiversity. On the other hand, it can help repair polluted and degraded ecosystems. For example, editing genes in certain plants to enable them to hyperaccumulate heavy metals can be used for phytoremediation of soil heavy metal pollution, or enhancing plants’ tolerance to harsh environments can be applied to vegetation restoration in mine waste lands, desertified lands, etc., promoting the restoration and reconstruction of ecosystems.

Gene-editing technology offers considerable potential for alleviating biotic and abiotic stress. Despite the promising potential of gene-editing technology, it faces various challenges. Off-target effects constitute a significant concern that requires immediate attention. Moreover, achieving effective and sustained gene transfer and editing requires ongoing research to improve editing efficacy and success rates. Regulatory policies for gene-edited crops differ across countries and sectors. To ensure the safe and healthy growth of the technology while preventing potential problems, there needs to be greater international communication and cooperation to create a unified, scientific, and acceptable regulatory framework. Promoting long-term agricultural growth, guaranteeing the world’s food supply, and safeguarding the ecological environment are all crucial to ensuring a better future for humanity. Technological advancements, along with their consistently expanding applications and effective responses to challenges, will surely play an indispensable role in these endeavors.

## Data Availability

No new data were created or analyzed in this study.

## Abbreviations

CRISPR: Clustered Regularly Interspaced Short Palindromic Repeats; gRNA: guide RNA; *dsx*: doublesex; *SUT*: sucrose transporter; *OsHMA*: *Oryza sativa* heavy metal ATPase; *OsNRAM*: *Oryza sativa* natural resistance-associated macrophage protein; *ZmHAK*: *Zea mays* high-affinity K^+^ transporter.
